# Supplementary material for: Viromes of Antarctic fish resemble the diversity found at lower latitudes
Source: Virus Evol. 2024 Jul 11;10(1):veae050. doi: 10.1093/ve/veae050 (PMC11282168; doi:10.1093/ve/veae050)
Supplement: veae050_Supp [file veae050_supp.zip › suppl_data/Supplementary Legends.docx]

**Supplementary Figure Legends**

**Supplementary Figure 1. Correlation between host groups sampled and number of viruses uncovered across fish viromes studies.** Plots showing the correlation between individuals sampled (left) and host groups sampled (right) and number of virus taxonomic groups (top) and unique viruses (bottom) uncovered in fish viromes studies between 2021 and 2024. R^2^ values were calculated using Spearman correlation tests.

**Supplementary Figure 2. Genome organisations of *Trematomus* host viruses.** Trematomus arenavirus (ambisense single-stranded RNA) genome, including the L, M, and S segments (top). Circularised Ross Sea Perciformes nackednavirus (reverse-transcribed RNA) genome (bottom). Host species are shown by fish illustrations.

**Supplementary Figure 3. *Exploring the nucleoprotein (NP) of Trematomus arenavirus*.** A) Comparison of the topology in maximum likelihood trees of the translated ORFs containing the NP core (left) and exonuclease domain (right) of the NP in the novel fish host aligned with full nucleoproteins of a representative range of viruses from the *Arenaviridae*. Both domains fall within the *Antennavirus* genus. Predicted structures of the NP core (blue, left) and exonuclease domain (orange, right) are shown in grey dotted boxes. B) Read coverage across the NP from Trematomus arenavirus in *Trematomus loennbergii.* Read coverage of the NP gene was consistent across the sequence (top). The division of the NP into two ORFs may have arisen due to a frameshift mutation as the two ORFs are translated in two different reading frames (bottom).

**Supplementary Figure 4. *Arenavirus* nucleoprotein structural diversity.** ColabFold-predicted nucleoprotein structures of arenavirus NPs from fish, reptilian, and mammalian hosts. Proteins are orientated with the exonuclease domain on top of the nucleoprotein core. Structures noted with an asterisk symbol (*) indicate structures based on incomplete coding sequences.

**Supplementary Figure 5. Host genus influences fish viromes but host order and body weight do not.** Non-metric multidimensional scaling (NMDS) plots of fish viromes coloured by body mass (left), host genus (middle), and host order (right). The effect of host genus on virome composition was significant (PERMANOVA p = 0.013) but the effects of host order (p = 0.205) and body mass (p = 0.376) were not.
