# Supplementary material for: Viromes of Antarctic fish resemble the diversity found at lower latitudes
Source: Virus Evol. 2024 Jul 11;10(1):veae050. doi: 10.1093/ve/veae050 (PMC11282168; doi:10.1093/ve/veae050)
Supplement: veae050_Supp [file veae050_supp.zip › suppl_data/Supplementary_Figure_1.pdf]

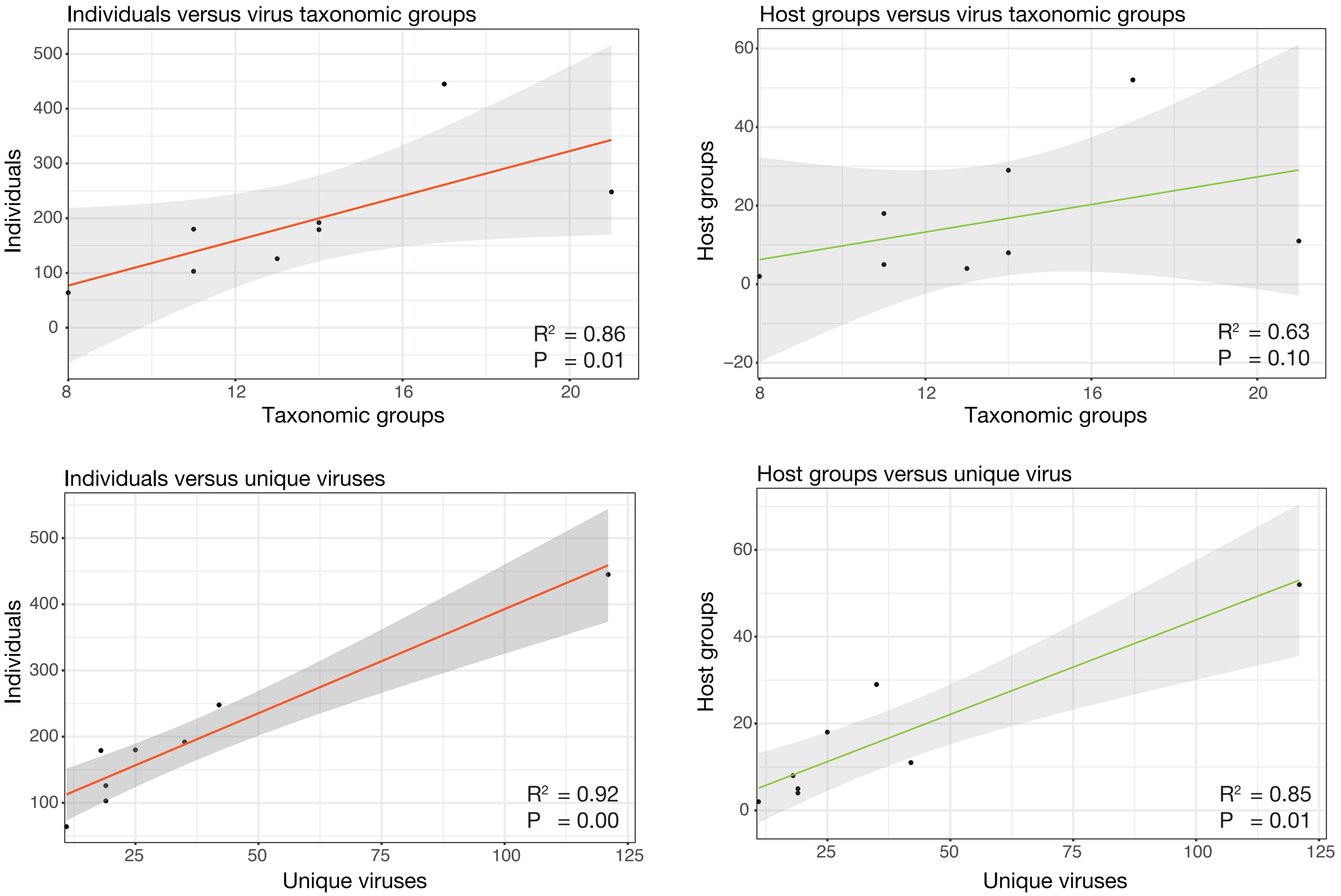
Individuals versus virus taxonomic groups
Individuals
500
400
300
200
100
0
8
12
16
20
Taxonomic groups
R^2 = 0.86
P = 0.01
Host groups versus virus taxonomic groups
Host groups
60
40
20
0
−20
8
12
16
20
Taxonomic groups
R^2 = 0.63
P = 0.10
Individuals versus unique viruses
Individuals
500
400
300
200
100
25
50
75
100
125
Unique viruses
R^2 = 0.92
P = 0.00
Host groups versus unique virus
Host groups
60
40
20
0
25
50
75
100
125
Unique viruses
R^2 = 0.85
P = 0.01
