# Supplementary material for: Viromes of Antarctic fish resemble the diversity found at lower latitudes
Source: Virus Evol. 2024 Jul 11;10(1):veae050. doi: 10.1093/ve/veae050 (PMC11282168; doi:10.1093/ve/veae050)
Supplement: veae050_Supp [file veae050_supp.zip › suppl_data/Supplementary_Figure_2.pdf]

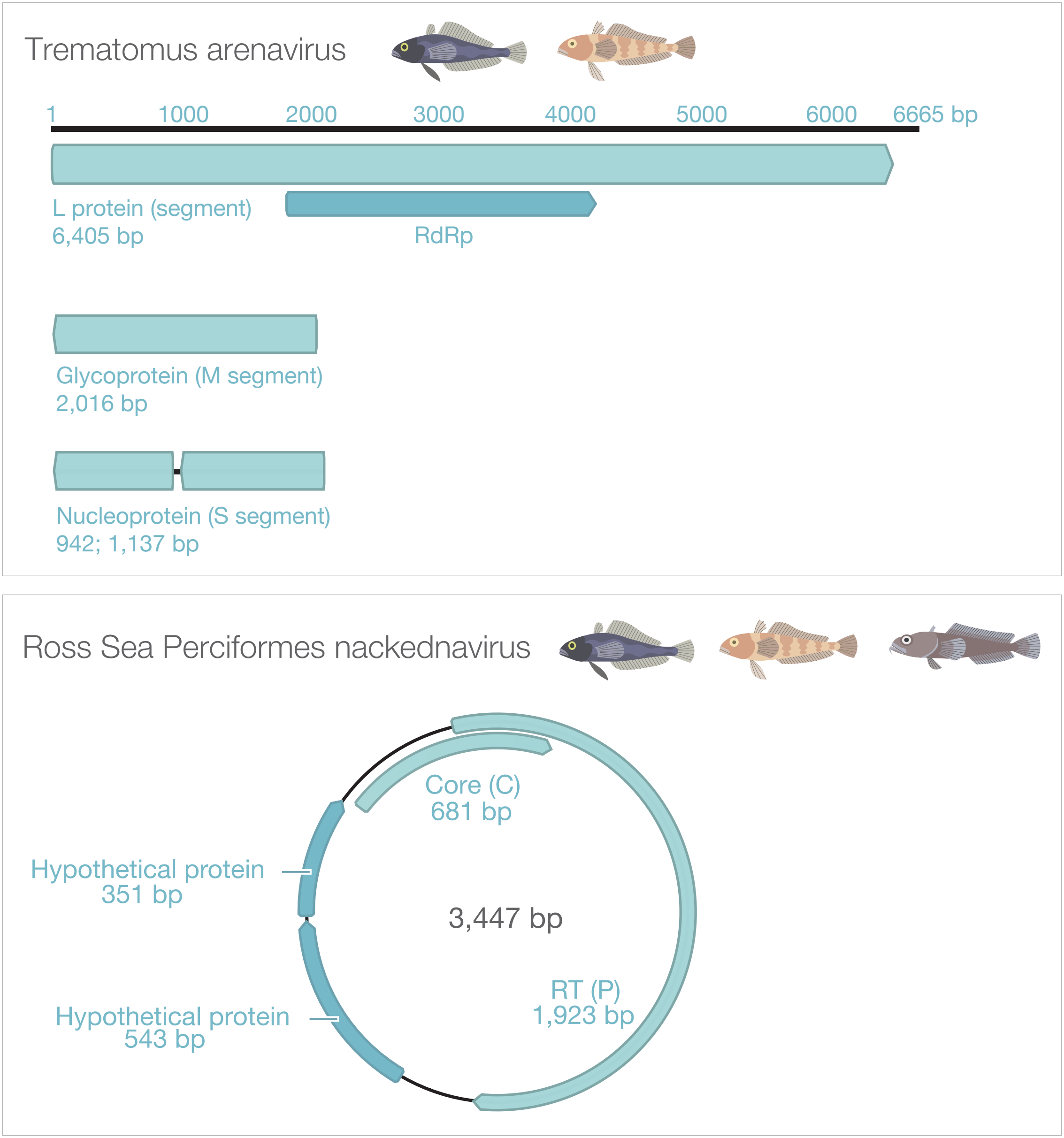

Trematomus arenavirus
1
1000
2000
3000
4000
5000
6000
6665 bp
L protein (segment)
6,405 bp
RdRp
Glycoprotein (M segment)
2,016 bp
Nucleoprotein (S segment)
942; 1,137 bp
Ross Sea Perciformes nackednavirus
Core (C)
681 bp
Hypothetical protein
351 bp
3,447 bp
RT (P)
1,923 bp
Hypothetical protein
543 bp
