# Supplementary material for: Viromes of Antarctic fish resemble the diversity found at lower latitudes
Source: Virus Evol. 2024 Jul 11;10(1):veae050. doi: 10.1093/ve/veae050 (PMC11282168; doi:10.1093/ve/veae050)
Supplement: veae050_Supp [file veae050_supp.zip › suppl_data/Supplementary_Figure_4.pdf]

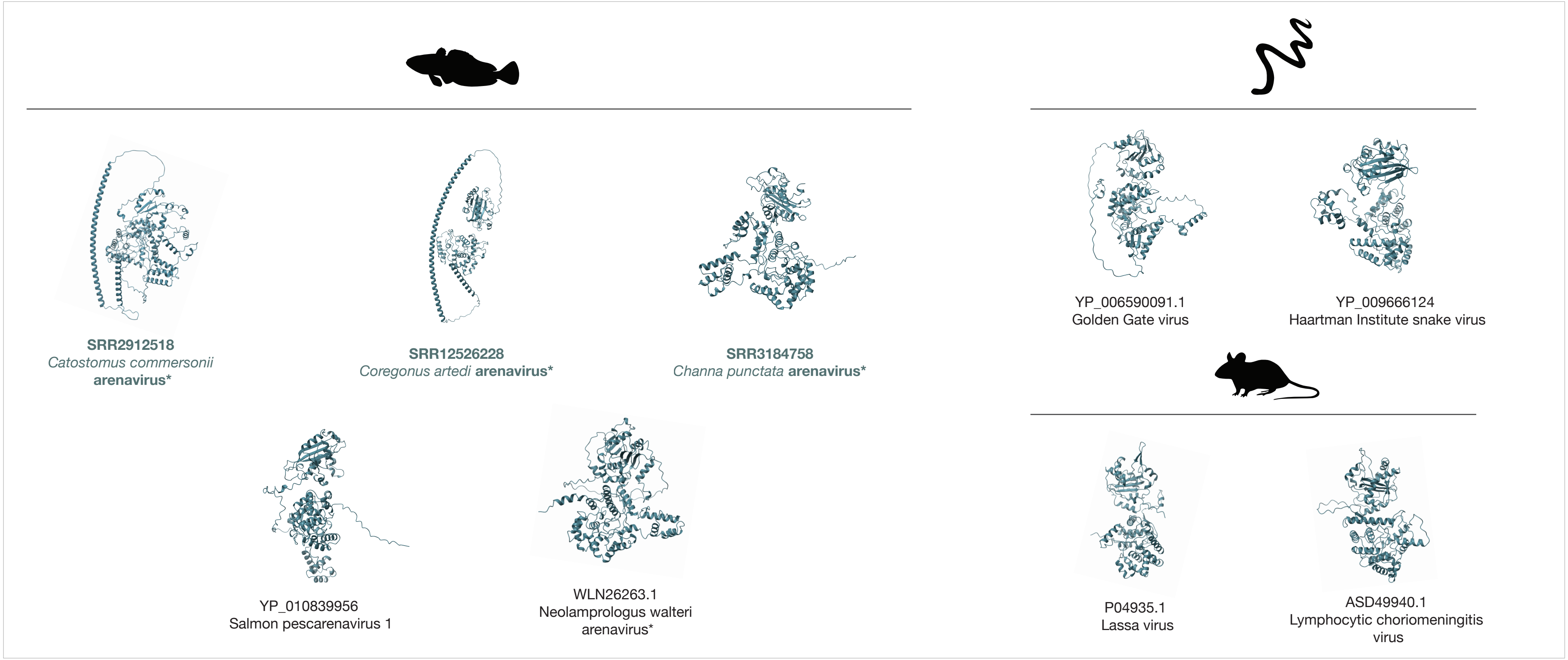
SRR2912518
Catostomus commersonii arenavirus*
SRR12526228
Coregonus artedi arenavirus*
SRR3184758
Channa punctata arenavirus*
YP_010839956
Salmon pescarenavirus 1
WLN26263.1
Neolamprologus walteri arenavirus*
YP_006590091.1
Golden Gate virus
YP_009666124
Haartman Institute snake virus
P04935.1
Lassa virus
ASD49940.1
Lymphocytic choriomeningitis virus
