# Supplementary figures and images for: Viromes of Antarctic fish resemble the diversity found at lower latitudes
Source: Virus Evol. 2024 Jul 11;10(1):veae050. doi: 10.1093/ve/veae050 (PMC11282168; doi:10.1093/ve/veae050)

Nucleoprotein core

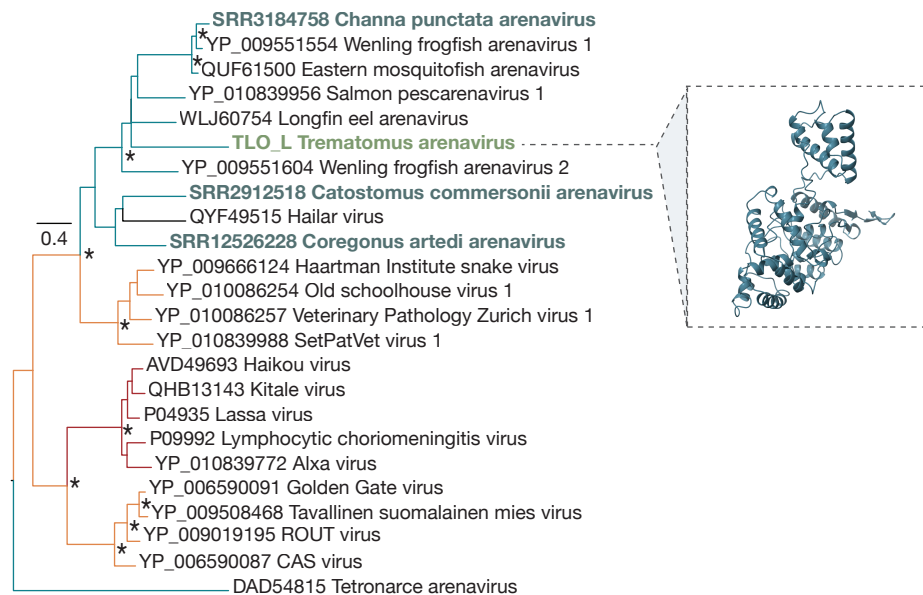

## Exonuclease domain

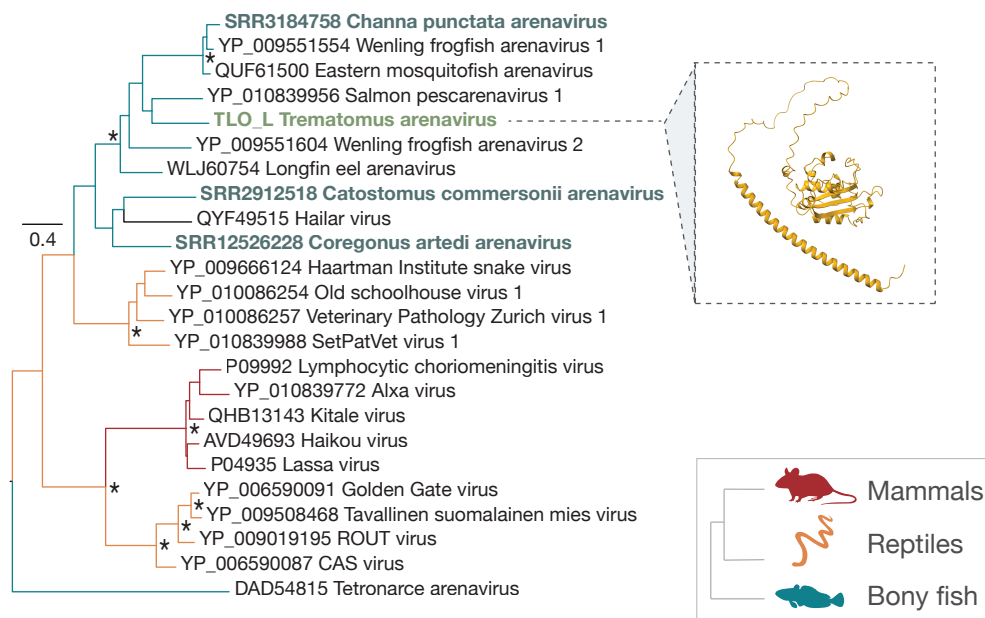

B

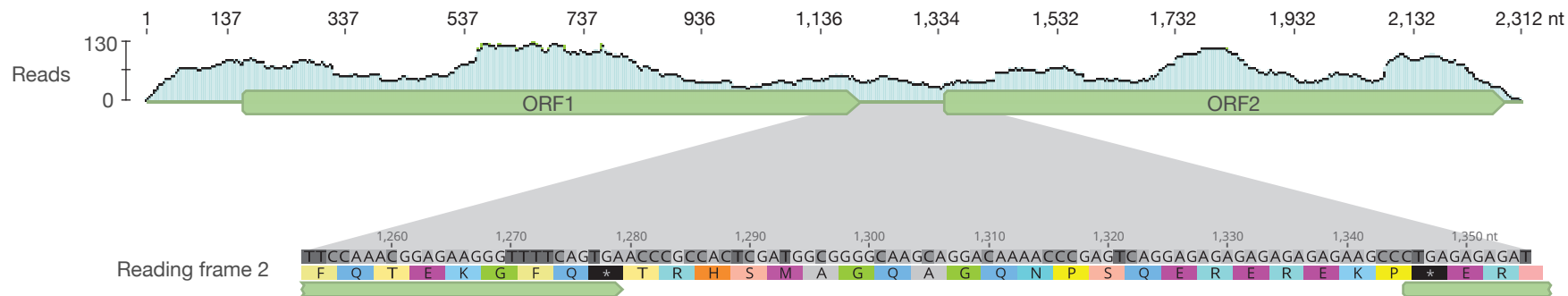

Supplement: veae050_Supp [file veae050_supp.zip › suppl_data/Supplementary_Figure_3.pdf]

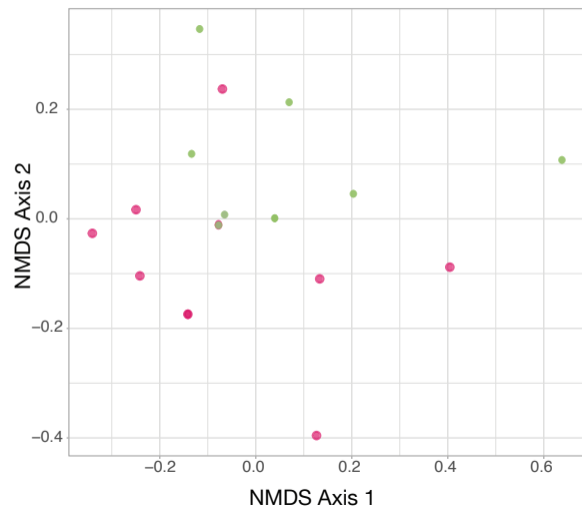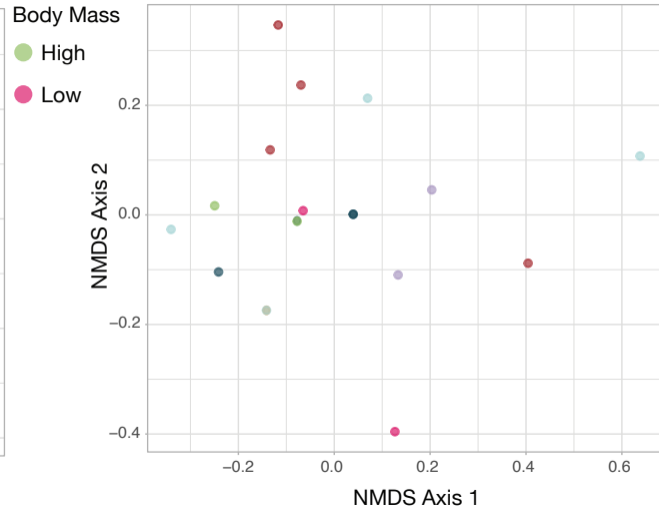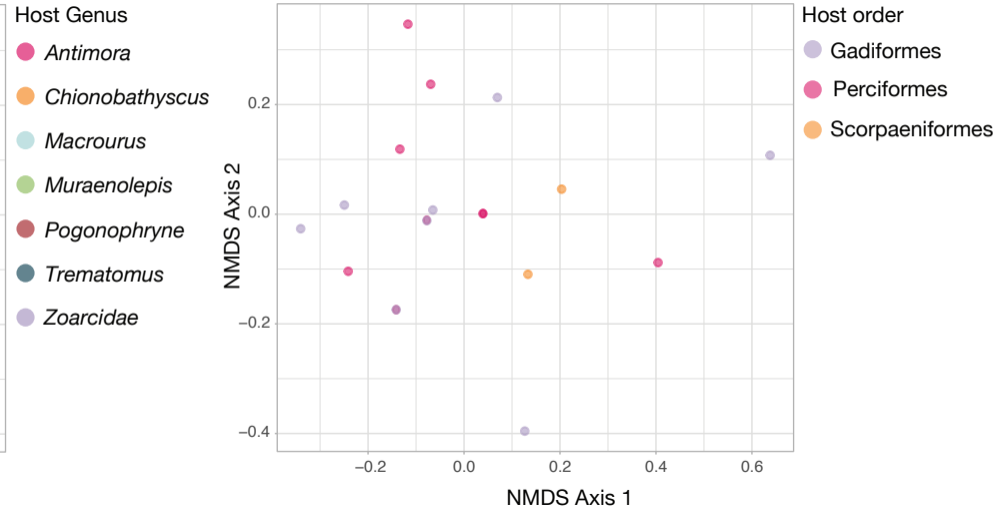

Supplement: veae050_Supp [file veae050_supp.zip › suppl_data/Supplementary_Figure_5.pdf]
